# Ivacaftor: Five‐year outcomes in the West of Scotland cystic fibrosis population

**DOI:** 10.1111/crj.13602

**Published:** 2023-03-20

**Authors:** Yasmin Al‐Din, Carol Dryden, Gordon MacGregor, David Young, Cristina Coelho

**Affiliations:** ^1^ Department of Pharmacy Queen Elizabeth University Hospital, NHS Greater Glasgow and Clyde Glasgow UK; ^2^ Department of Paediatrics University Hospital Wishaw, NHS Lanarkshire Wishaw UK; ^3^ Department of Respiratory Medicine Queen Elizabeth University Hospital, NHS Greater Glasgow and Clyde Glasgow UK; ^4^ Department of Mathematics and Statistics University of Strathclyde Glasgow UK; ^5^ Department of Pharmacy Glasgow Royal Infirmary, NHS Greater Glasgow and Clyde Glasgow UK

**Keywords:** cystic fibrosis, cystic fibrosis transmembrane conductance regulator, G551D mutation, ivacaftor, pulmonary, real world

## Abstract

**Introduction:**

Ivacaftor has shown to be effective in patients with cystic fibrosis (CF) with a G551D mutation.

**Objectives:**

This work aims to evaluate ivacaftor's effectiveness and safety in the real world, over 5 years, in the West of Scotland CF population.

**Methods:**

We evaluated ivacaftor's effect on pulmonary function, body mass index (BMI), hospital bed occupancy, and adverse effects in patients ≥6 years with at least one G551D mutation.

**Results:**

Statistically significant increases from baseline were observed in mean per cent predicted forced expiratory volume in 1 s (FEV_1_) at year 1 (which was maintained at years 2 and 5) and BMI over 5 years in our adolescent/adult cohort. Improvements were observed in per cent predicted FEV_1_ within the paediatric cohort with a suggestion of a plateau effect. The increase in paediatric BMI *z*‐score was nonstatistically significant. There was a reduction in the number of pulmonary exacerbations requiring intravenous antibiotics and hospital bed occupancy. Ivacaftor was well tolerated.

**Conclusion:**

Ivacaftor was effective in our population.

## Graphical abstract

Ivacaftor's effectiveness and safety was evaluated in the real world, over 5 years, in patients with at least one G551D mutation within the West of Scotland. Findings include the following: Improvement in mean per cent predicted forced expiratory volume in 1 s (FEV_1_), sustained increase in body mass index, decreased intravenous antibiotic use from baseline, reduced hospital bed occupancy from baseline, and good safety profile.

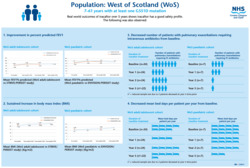

## 1 INTRODUCTION

Ivacaftor is licensed for the treatment of cystic fibrosis (CF) caused by specific gene mutations including G551D.^1^ Efficacy was reported in two randomised, double blind, placebo controlled trials in adolescent/adult ≥12 years (STRIVE), in paediatric patients ≥6 years (ENVISION), and one open label follow‐up study of participants from both trials (PERSIST).^2^, ^3^, ^4^ STRIVE and ENVISION showed improvement in lung function, pulmonary exacerbations, and other clinical outcomes at 48 weeks, compared with placebo.^2^, ^3^ In PERSIST, improvement was maintained at 144 weeks with no additional safety concerns.^4^ We report 5‐year real‐world outcomes of West of Scotland (WoS) patients on ivacaftor.

## 2 MATERIALS AND METHODS

Patients ≥6 years under the care of the CF adolescent/adult and paediatric teams in National Health Service Greater Glasgow and Clyde (NHSGGC) and CF paediatric team in NHS Lanarkshire with at least one G551D mutation treated with ivacaftor since 2012/2013 were identified. Data were collected retrospectively in 2018, at four time‐points (baseline and at year 1, 2, and 5 post‐treatment) using electronic patient records for forced expiratory volume in 1 s (FEV_1_) and weight using the value closest to the 1 year mark, height, age, gender, smoking status, CFTR mutation class, date of treatment initiation, treatment interruption and reasons, hospital admissions related to pulmonary exacerbations requiring treatment with intravenous (IV) antibiotics, liver function tests (LFTs), and adverse drug reactions (ADRs) reported in clinical letters as suspected to be ivacaftor related. Body mass index (BMI) was calculated. In the paediatric cohort, standard deviation scores (SDS or *z*‐scores) were calculated using the LMS method of Cole et al for weight, height and BMI, using UK 1990 growth reference data, based on age and gender.^5^, ^6^ The Global Lung Function Initiative (GLI) equation was used to calculate per cent predicted FEV_1_. Data were anonymised and entered into an Excel spreadsheet for analysis. All data were tested for normality using the Anderson–Darling test; parametric tests were subsequently performed. Changes over time were investigated using repeated measures analysis of variance. If differences were found, they were analysed using pairwise comparisons between time‐point with a Bonferroni correction. Analyses were performed using Minitab (version 18) at a 5% significance level.

## 3 RESULTS

Thirty‐two patients were identified. One patient was excluded due to missing data at three data collection time‐points including baseline. Two patients deceased resulting in a reduced sample size at year 5. Table 1 details WoS cohorts' baseline characteristics.

**TABLE 1:** Patient demographics and baseline characteristics.

	Adolescent/adult WoS cohort (*n* = 24)	Paediatric WoS cohort (*n* = 7)	Adolescent/adult STRIVE (*n* ^a^ = 83)	Paediatric ENVISION (*n* ^a^ = 26)
Gender *n* (%)	14 males (58%) 10 females (42%)	7 females (100%)	39 males (47%) 44 females (53%)	9 males (35%) 17 females (65%)
Age (years, mean [range])	24.3 (13–61)	8.4 (7–10)	26.2 (12–53)	8.9 (6–11)
Baseline FEV_1_ (% predicted, mean [range])	67.3 (17.8–107.7)	87.6 (78.0–103.0)	63.5 (37.3–98.2)	84.7 (52.4–133.8)
Baseline weight (kg, mean [range])	61.3 (48.0–89.4)	27.0 (18.3–34.0)	61.7 (30.2–107.2)	31.8 (18.8–62.6)
Baseline paediatric weight *z*‐score (range)	Not applicable	−0.16 (−1.85–1.10)	Not applicable	z‐score not reported
Baseline height (cm, mean [range])	169.8 (156–192)	130.7 (116–144)	167.7 (143–185)	134.9 (115–169)
Baseline paediatric height *z*‐score (range)	Not applicable	0.22 (−1.25–2.06)	Not applicable	z‐score not reported
Baseline BMI (kg/m^2^, mean [range])	21.1 (16.8–26.2)	15.7 (13.5–19.8)	21.7 (14.8–38.9)	17.1 (14.2–26.0)
Baseline paediatric BMI *z*‐score (range)	Not applicable	−0.41 (−1.52–1.52)	Not applicable	0.09 (−1.50–2.10)
Second gene mutation class *n* (%)	Class I 2 (8%) Class II 18 (75%) Class IV 2 (8%) Class V 1 (4%) Unknown 1 (4%)	Class I 0 (0%) Class II 6 (86%) Class III 0 (0%) Class IV 1 (14%) Class V 0 (0%)	Class I 10 (12%) Class II 70 (84%) Class IV 2 (2%) Unknown 1(1%)	Class II 18 (69%) No further detail reported

*Note*: Data from STRIVE or ENVISION were extracted from Davies et al.^2^ and Ramsey et al.^3^

^a^ Number of patients included in the ivacaftor group for STRIVE and ENVISION.

Statistically significant increases from baseline were observed in mean per cent predicted FEV_1_ at year 1 (which was maintained at years 2 and 5) and BMI over 5 years in our adolescent/adult cohort (Table 2). In the paediatric cohort, the statistically significant improvement in mean per cent predicted FEV_1_ observed in year 1 and 2 was not sustained in year 5. A nonstatistically significant increase in paediatric BMI *z*‐scores was observed over 5 years.

**TABLE 2:** Mean change from baseline in per cent predicted FEV_1_and BMI.

Duration of ivacaftor treatment	Mean change in per cent predicted FEV_1_ from baseline (% points) Mean (95% CI) (*P* value)	Mean change in BMI from baseline (kg/m^2^) Mean (95% CI) (*P* value) Mean paediatric *z*‐score where applicable
**WoS adolescent/adult cohort**	**FEV**_**1**_ ***P* < 0.001**	**BMI *P* < 0.001**
Year 1 (*n* = 24)	7.5 (3.3, 11.8) (*P* < 0.001)	1.7 (0.6, 2.8) (*P* = 0.001)
Year 2 (*n* = 24)	6.1 (1.8, 10.4) (*P* = 0.002)	1.8 (0.8, 2.9) (*P* < 0.001)
Year 5 (*n* = 22)^a^	7.4 (3.0, 11.8) (*P* < 0.001)	2.7 (1.6, 3.8) (*P* < 0.001)
**WoS paediatric cohort**	**FEV**_**1**_ ***P* = 0.006**	**BMI *P* = 0.001** ***BMI z‐score P = 0.467***
Year 1 (*n* = 7)	12.8 (4.4, 21.3) (*P* = 0.003)	1.0 (−0.8, 2.9) (*P* = 0.455) *0.33 (−0.36, 1.02) (P = 0.672)*
Year 2 (*n* = 7)	9.2 (0.8, 17.7) (*P* = 0.036)	1.8 (−0.01, 3.7) (*P* = 0.052) *0.34 (−0.35, 1.03) (P = 0.622)*
Year 5 (*n* = 7)	4.4 (−4.1, 12.8) (*P* = 0.607)	3.4 (1.6, 5.3) (*P* < 0.001) *0.079 (−0.61, 0.77) (P = 1)*
**Pivotal adolescent/adult trials**		
Year 1 (STRIVE) (*n* = 83)^b^	10.1 (*P* value not reported)	Mean BMI change not reported
Year 2 (PERSIST) (*n* = 74)^c^	9.1 (*P* value not reported)	1.0 (*P* value not reported)
**Pivotal paediatric trials**		
Year 1 (ENVISION) (*n* = 26)^b^	10.7 (*P* value not reported)	Mean BMI change not reported *0.45 (P < 0.001)*
Year 2 (PERSIST) (*n* = 25)^c^	9.0 (*P* value not reported)	0.32 (*P* value not reported)

*Note*: Data from STRIVE, ENVISION, and PERSIST were extracted from previous works.^2^, ^3^, ^4^

^a^ Reduced sample size because two patients died at year 5 time‐point.

^b^ Number of patients included in the ivacaftor group for STRIVE or ENVISION.

^c^ Number of patients included in the ivacaftor group for STRIVE/PERSIST or ENVISION/PERSIST.

The number of patients and episodes of pulmonary exacerbations requiring IV antibiotics and time spent in hospital reduced from baseline over 5 years in both cohorts (Table 3).

**TABLE 3:** Pulmonary exacerbations requiring hospital admission for West of Scotland patients on ivacaftor.

Duration of ivacaftor treatment	Number of patients with pulmonary exacerbations requiring IV antibiotics (%)	Number of episodes of pulmonary exacerbations requiring IV antibiotics	Total duration of hospital admission (days)	Mean bed days per patient per year
**WoS adolescent/adult cohort**				
Baseline (*n* = 24)	8 (33%)	20	165	7
Year 1 (*n* = 24)	4 (17%)	9	71	3
Year 2 (*n* = 24)	5 (21%)	12	97	4
Year 5 (*n* = 22)^a^	5 (23%)	10	99	5
**WoS paediatric cohort**				
Baseline (*n* = 7)	4 (57%)	6	84	12
Year 1 (*n* = 7)	3 (43%)	4	56	8
Year 2 (*n* = 7)	1 (14%)	2	28	4
Year 5 (*n* = 7)	1 (14%)	1	14	2

^a^ Reduced sample size because two patients died at year 5 time‐point.

Ivacaftor interruptions were due to nonadherence and pregnancy (*n* = 4), possible ADRs (*n* = 2: eosinophilia and right upper quadrant discomfort), deranged LFTs (*n* = 1). One ADR (diarrhoea) led to ivacaftor dose reduction. No discontinuations to ivacaftor occurred. Other possible reported reactions included rhinitis and transient unilateral ear swelling. Two patients experienced deranged LFTs; these were not specifically attributed to ivacaftor.

## 4 DISCUSSION

Improvement in mean per cent predicted FEV_1_ was observed. The improvement of 7.5% from baseline within the adolescent/adult WoS cohort at year 1 and 6.1% at year 2 was statistically significant but lower than the improvement of 10.1% in STRIVE at year 1 and 9.1% in PERSIST at year 2.^2^, ^4^ This difference may be due to treatment in the real world and inclusion of four patients with a per cent predicted FEV_1_ <40% at baseline, an exclusion criteria in STRIVE. Within our paediatric cohort, the improvement from baseline of 12.8% at year 1 was statistically significant and greater than the improvement of 10.7% in ENVISION.^3^ The improvement of 9.2% at year 2 was statistically significant and approximately equivalent to the improvement of 9% in PERSIST.^4^ Dryden et al reported a lower mean change in per cent predicted FEV_1_ at year 1, in paediatric patients compared with clinical trials.^7^ Hurbet et al found a lower increase in per cent predicted FEV_1_ from baseline at year 1 and 2, compared with clinical trials in patients ≥6 years with the adolescent group showing the highest response.^8^ At year 5, an improvement in per cent predicted FEV_1_, from baseline was seen in both WoS cohorts; this was not statistically significant in the paediatric cohort possibly due to our small sample size. Two adolescent/adult patients deceased prior to year 5, which may have impacted our results at year 5. Mitchell et al showed that after 3 months of treatment in adults FEV_1_ declined and was not significantly different from baseline at year 5.^9^ Robson et al reported improvement at year 5 in paediatrics compared with year 1.^10^

A statistically significant increase in WoS adolescent/adult BMI was observed. The increase in paediatric BMI *z*‐score was nonstatistically significant, possibly due to the small sample size. At year 2, the mean change in BMI from baseline was greater by 0.8 and 1.5 kg/m^2^ within our adolescent/adult and paediatric cohorts, respectively, compared with PERSIST.^4^ Increased BMI may be desirable; however, care is needed to avoid associated adverse outcomes.

Over 5 years, there was a decrease in the number of patients and pulmonary exacerbations requiring IV antibiotics, and duration of hospital admissions. Within the adolescent/adult cohort, the largest reduction, from baseline, was at year 1. Mitchell et al showed a significant sustained reduction in IV antibiotic use, over 5 years, in adults.^9^ Our paediatric cohort showed a sustained decrease over 5 years. This is important as pulmonary exacerbations can accelerate the rate of lung function decline.

Adherence to ivacaftor, in our cohorts, was encouraged through dialogue and delivery monitoring. There was no suggestion of nonadherence in our paediatric cohort. The literature reports non‐adherence rates of 61–80%.^11^, ^12^

Limitations include the following: Small sample size, gender bias (5 year paediatric male data was unavailable), and lack of a comparator group.

## 5 CONCLUSIONS

Our findings support ivacaftor's effectiveness.^13^ Work is needed to assess quality of life and effectiveness with a comparator group.

## AUTHOR CONTRIBUTIONS

Yasmin Al‐Din designed the research study, collected the data, analysed the data, and wrote paper. Dr Carol Dryden designed the research study, collected the data, analysed the data, and contributed to the content of the paper. Dr Gordon Macgregor designed the research study and contributed to the content of the paper. Dr David Young designed the statistical analysis to the research study and provided the statistics to the research study. Cristina Coelho designed the research study and contributed to the content of the paper.

## CONFLICT OF INTEREST STATEMENT

Dr Dryden received personal fees from a Vertex Advisory Board meeting regarding a different drug, that is, outside the submitted work. Dr MacGregor has attended advisory boards and been a principle and chief investigator on a number of Vertex trials.

## ETHICS STATEMENT

Ethics approval was not required in accordance with NHS Research Ethics Service guidance.

## Data Availability

Research data are not shared.

## References

[1] Barry P , Donaldson A , Jones A . Ivacaftor for cystic fibrosis. BMJ. 2018;361:K1783. 29773589 10.1136/bmj.k1783

[2] Davies JC , Wainwright CE , Canny GJ , et al. Efficacy and safety of ivacaftor in patients aged 6 to 11 years with cystic fibrosis with a G551D mutation. Am J Respir Crit Care Med. 2013;187(11):1219‐1225. doi:10.1164/rccm.201301-0153OC 23590265 PMC3734608

[3] Ramsey BW , Davies J , McElvaney NG , et al. A CFTR potentiator in patients with cystic fibrosis and the G551D mutation. N Engl J Med. 2011;365(18):1663‐1672. doi:10.1056/NEJMoa1105185 22047557 PMC3230303

[4] McKone EF , Borowitz D , Drevinek P , et al. Long‐term safety and efficacy of ivacaftor in patients with cystic fibrosis who have the Gly551Asp‐CFTR mutation: a phase 3, open‐label extension study (PERSIST). Lancet Respir Med. 2014;2(11):902‐910. doi:10.1016/S2213‐2600(14)70218‐8 25311995 10.1016/S2213-2600(14)70218-8

[5] Cole TJ , Freeman JV , Preece MA . Body mass index reference curves for the UK, 1990. Arch Dis Child. 1995;73(1):25‐29. doi:10.1136/adc.73.1.25 7639544 PMC1511150

[6] Freeman JV , Cole TJ , Chinn S , Jones PR , White EM , Preece MA . Cross sectional stature and weight reference curves for the UK, 1990. Arch Dis Child. 1995;73(1):17‐24. doi:10.1136/adc.73.1.17 7639543 PMC1511167

[7] Dryden C , Wilkinson J , Young D , Brooker RJ , Scottish Paediatric Cystic Fibrosis Managed Clinical Network (SPCFMCN) . The impact of 12 months treatment with ivacaftor on Scottish paediatric patients with cystic fibrosis with the G551D mutation: a review. Arch Dis Child. 2018;103(1):68‐70. doi:10.1136/archdischild-2015-310420 27288428

[8] Hubert D , Dehillotte C , Munck A , et al. Retrospective observational study of French patients with cystic fibrosis and a Gly551Asp‐CFTR mutation after 1 and 2 years of treatment with ivacaftor in a real‐world setting. J Cyst Fibros. 2018;17(1):89‐95. doi:10.1016/j.jcf.2017.07.001 28711222

[9] Mitchell RM , Jones AM , Barry PJ . Longitudinal effects of ivacaftor therapy in adults with the G551D mutation—a 5‐year study. J Cyst Fibros. 2019;18(Suppl 1):S21‐S22 [abstract ‐ WS12‐1]. doi:10.1016/S1569-1993(19)30185-7

[10] Robson EA , Feltbower R , Lee T . Real world ivacaftor efficacy in children: five years on. J Cyst Fibros. 2019;18(Suppl 1):S128 [abstract – P252]. doi:10.1016/S1569-1993(19)30545-4

[11] Suthoff ED , Bonafede M , Limone B , O'Callaghan L , Sawicki GS , Wagener JS . Healthcare resource utilization associated with ivacaftor use in patients with cystic fibrosis. J Med Econ. 2016;19(9):845‐851. doi:10.1080/13696998.2016.1178125 27074519

[12] Siracusa CM , Ryan J , Burns L , et al. Electronic monitoring reveals highly variable adherence patterns in patients prescribed ivacaftor. J Cyst Fibros. 2015;14(5):621‐626. doi:10.1016/j.jcf.2015.05.009 26074007 PMC4567928

[13] Volkova N , Moy K , Evans J , et al. Disease progression in patients with cystic fibrosis treated with ivacaftor: data from national US and UK registries. J Cyst Fibros. 2020;19(1):68‐79. doi:10.1016/j.jcf.2019.05.015 31196670

